# A Novel Noninvasive Screening Tool for Dry Eye Disease

**DOI:** 10.3390/diagnostics14121209

**Published:** 2024-06-07

**Authors:** Sabrina Vaccaro, Massimiliano Borselli, Giovanni Scalia, Costanza Rossi, Mario Damiano Toro, Robert Rejdak, Marco Pellegrini, Vincenzo Scorcia, Giuseppe Giannaccare

**Affiliations:** 1 Department of Ophthalmology, University Magna Græcia of Catanzaro, Viale Europa, 88100 Catanzaro, Italy; sabrina_vaccaro@libero.it (S.V.); mborselli93@gmail.com (M.B.); drscalia.giovanni@gmail.com (G.S.); costanzarossi9@gmail.com (C.R.); vscorcia@unicz.it (V.S.); 2 Eye Clinic, Public Health Department, University of Naples Federico II, Via S. Pansini 5, 80133 Naples, Italy; mariodamiano.toro@unina.it; 3 Department of General and Pediatric Ophthalmology, Medical University of Lublin, 20-079 Lublin, Poland; robert.rejdak@umlub.pl; 4 Department of Translational Medicine, University of Ferrara, 44121 Ferrara, Italy; marco.pellegrini@hotmail.it; 5 Department of Ophthalmology, Ospedali Privati Forlì “Villa Igea”, 47122 Forlì, Italy; 6 Eye Clinic, Department of Surgical Sciences, University of Cagliari, Via Università 40, 09124 Cagliari, Italy

**Keywords:** dry eye, diagnosis, noninvasive diagnosis, device, screening

## Abstract

Purpose: To assess the feasibility and the diagnostic accuracy of the new tool, DEvice© (AI, Rome, Italy), for screening patients with dry eye disease (DED). Methods: This study was performed at the University Magna Græcia of Catanzaro. Enrolled patients were classified as affected by DED (group 1) or not (group 2) using an already validated tool (Keratograph 5M, Oculus, Germany), evaluating the noninvasive keratograph breakup time (NIKBUT), tear meniscus height (TMH), meibomian gland loss (MGL), and bulbar redness. All the patients were then examined by means of DEvice©, which allowed the measurement of the relative humidity (RH) and temperature of the ocular surface. Symptoms were scored using the Ocular Surface Disease Index (OSDI) questionnaire. Results: Overall, 40 patients (17 males and 23 females, mean age 38.0 ± 17.1 years) were included: of these, 20 belonged to group 1 and the remaining 20 to group 2. Using Keratograph 5M, significant differences between groups 1 and 2 were detected for NIKBUT-first (respectively, 4.97 ± 1.85 vs. 13.95 ± 4.8 s; *p* < 0.0001) and for NIKBUT-average (10.55 ± 4.39 vs. 15.96 ± 4.08 s; *p* = 0.0003). No statistically significant changes were detected for TMH (*p* = 0.565), MGL (*p* = 0.051), and bulbar redness (*p* = 0.687). Using Device©, a statistically significant higher value of RH was found in group 1 compared to group 2 (respectively, 85.93 ± 10.63 vs. 73.05 ± 12.84%; *p* = 0.0049). A statistically significant correlation was found between RH and OSDI (r = 0.406; *p* = 0.009). The value RH showed a discriminating power to detect DED with an AUC = 0.782 (standard error 0.07264; 95% CI 0.6401–0.9249; *p* = 0.0022). Conclusions: The DEvice© can effectively discriminate DED patients from healthy subjects. The parameter RH showed good sensitivity, making this tool ideal for a fast and noninvasive DED screening.

## 1. Introduction

Dry eye disease (DED) is a common disorder of the ocular surface affecting millions of people worldwide, characterized by a loss of homeostasis of the tear film and accompanied by ocular discomfort symptoms [1,2]. The symptoms reported by patients include burning, foreign body sensation, ocular discomfort, dryness, irritation, grinding, scratching, sanding sensation, soreness, stinging, burning, itching, and eye tiredness, leading to ocular pain [1,2]. The impact of DED on patients is substantial, affecting their visual function, daily activities, and overall quality of life (QOL) [3]. A comprehensive assessment of the worldwide epidemiology showed that the prevalence varied from 5 to 50% according to diagnostic criteria employed and populations examined. Additionally, the frequency of the disease is expected to further rise because of the progressive aging of the population [4].

Dry eye is distinguished by an accelerated tear film breakage, reduction in tear volume, and heightened evaporation of tears [5]. According to the underlying mechanism, it is classified into two subtypes that can coexist in the same clinical picture: (i) evaporative DED, which occurs when the meibomian glands do not function properly, and (ii) aqueous deficiency DED, which occurs when the lacrimal glands do not produce enough tears [1]. The eye’s surface is protected by the tear film, consisting of three layers: the lipid layer from the meibomian glands, the aqueous layer from the lacrimal glands, and the mucin layer from goblet cells. The lower eyelid allows the deposit of the tear film in a so-called tear meniscus. The lacrimal punctum, located medially, allows for the drainage of tears. An effective mechanism of tear film generation and drainage helps prevent evaporation from the eye’s surface [6]. The evaporation of the aqueous component of the tear film and the subsequent instability cause tear hyperosmolarity, which is a key factor in DED. Hyperosmolarity can activate the inflammatory cascade, causing cellular damage by affecting conjunctival cells that make mucin. This can worsen tear film instability, creating an adverse cycle [7,8,9]. An osmolarity of 308 mOsm/L or lower is deemed normal [10]. For a better understanding in the context of DED, an osmolarity level of 316 mOsm/L is considered the dividing line between mild and moderate-to-severe dry eye conditions [11].

The diagnosis and monitoring of DED can be accomplished by employing a range of validated assessments collecting information about a patient’s signs and symptoms. In particular, the Ocular Surface Disease Index (OSDI) questionnaire is the most often used tool to score ocular discomfort symptoms. It is a valid instrument for quantifying the severity of DED and is preferred for its ability to rapidly assess the impact of the disease on patients’ QOL [12,13]. According to the TFOS DEWS II report, the diagnosis of DED requires the presence of symptoms in association with at least one pathological sign among reduced tear film stability, increased tear osmolarity, or damage to the ocular surface (cornea/conjunctiva) epithelium [1]. An important indicator of the ocular surface homeostasis in DED is pre-corneal film stability [5]. One frequently employed instrument for studying pre-corneal film stability in a non-invasive way is the Keratograph 5M (Oculus Optikgeräte GmbH, Wetzlar, Germany), with good repeatability and reproducibility [14]. The evaporation of the tear film is closely connected to the lipid layer’s stability and composition. Various individual elements, including eye position, interpalpebral surface area, humidity, lighting conditions, and temperature, will influence evaporation [15,16]. There is no device available for detecting tear film evaporation. Researchers have used homemade prototypes or adapted a dermatologic apparatus by adding a closed chamber around the eye [17]. Recently, there have been notable advances in the technology used for DED diagnosis, resulting in the development of numerous innovative imaging techniques and noninvasive devices [18,19]. These devices provide automated test results, eliminating observer bias. They are noninvasive and do not affect the outcomes of future tests, making them valuable tools for distinguishing between healthy individuals and those with or potentially at risk for DED [19].

Among these, DEvice© (AI, Rome, Italy) is a novel, cost-effective, portable prototype ocular hygrometer that accurately assesses the gaseous state of the tear film by detecting variations in humidity levels within a controlled setting. The sensor quantifies the relative humidity (RH) at a specific temperature. It is positioned within a cup and with the entire ocular surface system, comprising the enclosed environment where the measurements are conducted. A previous observational pilot study showed higher RH values in a small sample of patients with DED when compared to healthy controls [20]. The aim of the present study was to employ DEvice© for discriminating patients affected by DED from healthy subjects and to calculate its diagnostic accuracy for DED screening.

## 2. Materials and Methods

### 2.1. Study and Patients

In this prospective controlled study, consecutive patients with a confirmed diagnosis of DED attending the ocular surface office for control visits, and age- and sex-matched healthy subjects attending the general office for routine eye checkups were enrolled and examined at the tertiary referral center (Department of Ophthalmology, University of Magna Grecia, Catanzaro, Italy) between January and June 2023. The study was approved by the local Ethics Committee (Comitato Etico Regione Calabria Sezione Area Centro). A detailed informed consent for participation in the study was signed by all patients, in accordance with the 1964 Declaration of Helsinki. The exclusion criteria for both patients and controls were the following: presence of active ocular inflammation, recent ocular surgery (within 3 months) to avoid fluctuations in signs/symptoms of DED commonly present in the early postoperative period, history of contact lens wearing, and recent (within 1 month) use of anti-inflammatory eye drops (e.g., topical corticosteroids or cyclosporine). Patients and controls were evaluated by means of a validated noninvasive tool (Keratograph 5M, Oculus, Wetzlar, Germany, v. 2.15r2) to confirm the altered (patients with DED) or healthy (control subjects) status of the ocular surface. The inclusion criteria for patients with DED were noninvasive keratograph breakup time (NIKBUT) less than 10 s and pathological value of OSDI ≥ 13. NIKBUT-first, NIKBUT-average, tear meniscus height (TMH), meibomian gland loss (MGL), and bulbar redness were calculated. The NIKBUT was measured as the time in seconds between the last complete blink to the start of interruption in the placid rings reflected on the surface of the cornea, which is automatically recognized by the equipment. The instrument generated two measures for NIKBUT: the time until the tear film’s initial disruption (NIKBUT-first) and the average duration of all instances of rupture (NIKBUT-average) [21]. Photographs of the lower TMH were acquired and evaluated for every participant. The measurements were obtained by placing a ruler perpendicular to the edge of the lid at the central position with respect to the center of the pupil [21,22].

Infrared meibography was performed to assess the extent of gland insufficiency. The process included utilizing a grading system known as meiboscore, which classifies deficiencies on a scale ranging from 0 to 3. Grade 0 means no gland loss, grade 1 indicates up to 33% gland loss, grade 2 represents 33% to 66% gland loss, and grade 3 suggests 67% or more gland loss [23]. Measurements of bulbar redness were automatically acquired by the use of the Oculus Keratograph 5M software (v. 2.15r2). The R-Scan module automatically categorizes redness levels in the bulbar and limbal areas, identifies blood vessels in the conjunctiva, and scores redness levels using a grading system [24].

**Figure 1:**
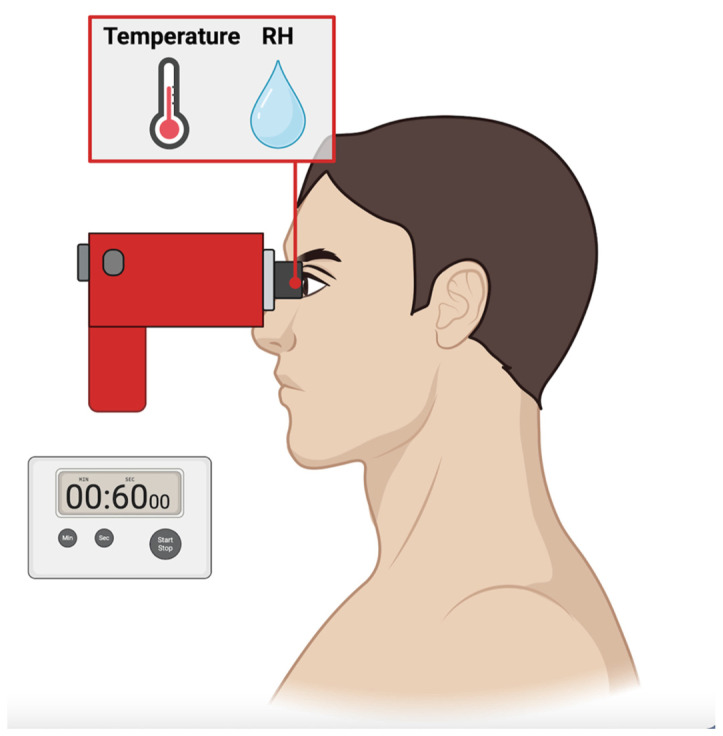
The DEvice© was placed by the operator on the orbital margins to measure parameters related to relative humidity (RH) and temperature.

Thirty minutes after the Keratograph assessment, all patients and controls were examined by means of DEvice© (AI, Rome, Italy), which allowed the measurement of RH and temperature in a closed microenvironment around the ocular surface. Technical details of the instrument along with its modality of use have been previously described [20]. Briefly, the DEvice© was positioned by the operator on the orbital margins of the patient, who was asked to avoid blinking during the assessment period (Figure 1). The operator positioned the device on the orbital margins and activated the measurement by pressing the button. The cup completely sealed the environment around the eye during the measurement period. After precisely 60 s, as determined by a timer, the operator pressed the button once more to conclude the collection of measurements.

RH and temperature values were obtained by calculating the average of all the measurements recorded. The RH value can be calculated using the formula: RH = ρω/ρs × 100%, where ρω represents the density of water vapor and ρs represents the density of saturated water vapor. The device was connected to a software program that provided the recorded data. An average of all recorded values was calculated.

The examinations with the two diagnostic tools were taken by the same experienced examiner (G.S.) in a dimly lit room during a single visit conducted between 10 am and 12 pm. The temperature was maintained between 21 and 24 °C and the humidity was held between 30 and 60%. The exam with the DEvice was repeated by another examiner after 15 min to assess intra-observer repeatability for RH parameters.

The OSDI questionnaire was used to evaluate symptoms of ocular pain, evaluating the symptoms reported by patients throughout the past week. The questionnaire consists of 12 items that evaluate 3 specific areas of symptomatology: vision-related function, ocular symptoms, and environmental triggers. Each response is assigned a score ranging from 0 to 4, where 0 represents “none of the time” and 4 represents “all of the time”. The cumulative total of all values will determine the OSDI score, which ranges from 0 to 100, where higher values indicate more severe disability. A score of 0–12 is normal, 13–22 indicates mild disease, 23–32 is moderate DED, and 33–100 suggests severe DED.

### 2.2. Statistical Analysis

Statistical analysis was performed using Prism version 9.5.0 (GraphPad Software Inc., San Diego, CA, USA) and Jamovi version 2.4.1.0 (The Jamovi Project, Sydney, Australia). Data were expressed as mean ± standard deviation (SD) if normally distributed, otherwise as median values with interquartile range (IQR). Anderson–Darling and Kolmogorov––Smirnov tests were applied to assess if data were normally distributed. The Student’s *t*-test or Mann–Whitney U-test were applied to compare variables when appropriate. The Pearson correlation test was used to evaluate the correlation of parameters. The accuracy of the RH parameter as a predictor of DED was evaluated by processing a receiver operating characteristic (ROC) curve. A *p*-value of less than 0.05 was considered as statistically significant. Bonferroni correction was used to control alpha error accumulation.

## 3. Results

**Figure 2:**
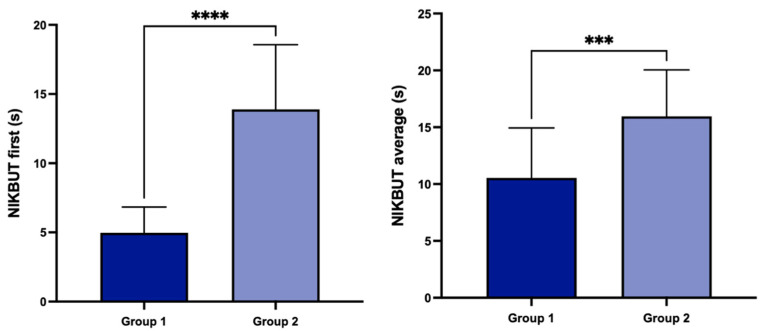
Noninvasive keratograph break-up time (NIKBUT) first and average in groups 1 and 2 (*** *p* < 0.001 and **** *p* < 0.0001).

The study included 40 eyes of 40 patients (17 males and 23 females, mean age 38 ± 17.14 years): 20 patients affected by DED (group 1) and 20 matched healthy subjects (group 2). There were no statistically significant differences between the two groups in terms of age and gender (*p* = 0.14 and *p* = 0.33, respectively). Using Keratograph 5M, all DED patients presented pathological values of at least one parameter, while all control subjects showed values within the normal range for all parameters. In detail, significant differences, according to the Bonferroni correction (*p* < 0.01), between groups 1 and 2 were detected for NIKBUT-first (respectively, 4.97 ± 1.85 vs. 13.95 ± 4.8 s; *p* < 0.0001) and for NIKBUT-average (respectively, 10.55 ± 4.39 vs. 15.96 ± 4.08 s; *p* = 0.0003; Figure 2).

No statistically significant differences were detected for TMH (0.27 ± 0.10 in group 1 vs. 0.26 ± 0.10 mm in group 2; *p* = 0.5656), bulbar redness (1.04 ± 0.34 in group 1 vs. 0.99 ± 0.45 in group 2; *p* = 0.6879), and MGL (1.00 [1.00–2.00] in group 1 and 1.00 [0.00–1.00] in group 2; *p* = 0.051).

**Figure 3:**
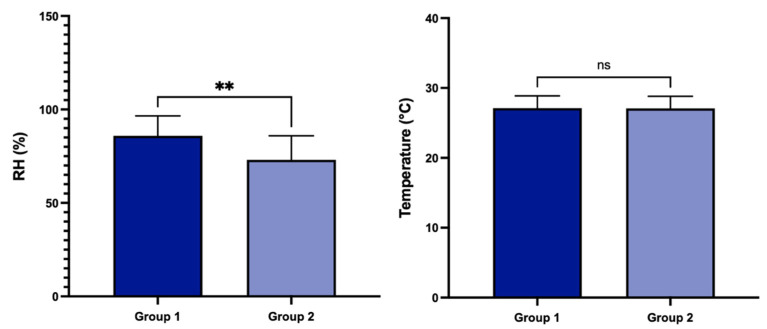
Values of relative humidity (RH) and temperature in groups 1 and 2 (** *p* < 0.01; ns, not significant).

Using DEvice©, a statistically significant higher value of RH, according to the Bonferroni correction (*p* < 0.025), was found in group 1 compared to group 2 (respectively, 85.93 ± 10.63 vs. 73.05 ± 12.84%; *p* = 0.0049). Conversely, mean values of temperature were not statistically significant between groups (27.12 ± 1.75 in group 1 vs. 27.08 ± 1.72 °C in group 2; *p* = 0.9318; Figure 3).

**Figure 4:**
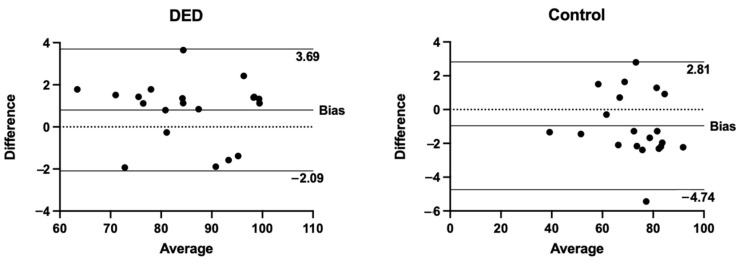
Analysis of agreement between two different measures by different examiners of RH values in the DED and control groups. The top and bottom lines show the 95% limits of agreement.

Bland–Altman analysis showed the intra-observer agreement of RH values for the DED and control groups (Figure 4).

**Figure 5:**
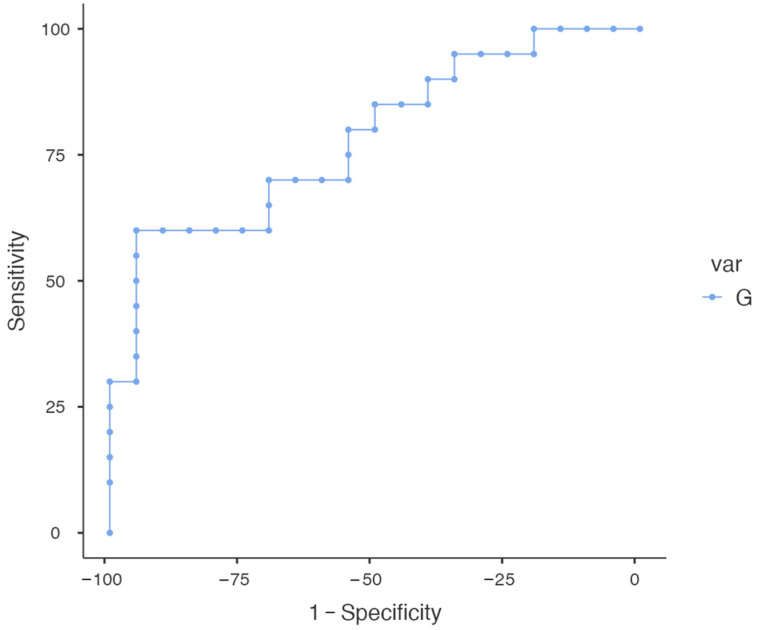
Receiver operating characteristic (ROC) curve of relative humidity (RH) for identifying patients with dry eye disease.

The area under the curve (AUC) was used to evaluate the accuracy of RH as a predictive value of DED as a continuous variable. The ROC curve of RH for identifying patients with DED is shown in Figure 5. A cutoff value of 84.88 was selected based on the Youden Index, resulting in a sensitivity of 60% and a specificity of 95%, with an AUC of 0.782 (standard error 0.07264; 95% CI 0.6401–0.9249; *p* = 0.0022).

Concerning ocular discomfort symptoms, there were statistically significant differences in the mean OSDI score between group 1 (42.15 ± 19.04) and group 2 (13.45 ± 7.61; *p* < 0.0001).

A statistically significant correlation, according to the Bonferroni correction (*p* < 0.01), was found between RH and OSDI (r = 0.406; *p* = 0.009), and a mild but not statistically significant correlation was found between RH and NIKBUT-first (r = −0.295; *p* = 0.065). Conversely, the other correlations of RH with TMH (r = −0.091; *p* = 0.577), NIKBUT-average (r = −0.101; *p* = 0.534), MGL (r = 0.129; *p* = 0.427), and bulbar redness (r = 0.046; *p* = 0.776) were not significant (Table 1).

## 4. Discussion

Dry eye is a prevalent disease that affects the ocular surface and impacts millions of individuals worldwide. The prevalence of this condition will persistently rise due to the increasing age of the population and changes in lifestyle [1,2,3,4]. Considering the high prevalence and the important clinical implications, there has been a significant increase in research focused on developing and validating novel devices for the noninvasive screening and diagnosis of DED [18,19]. The present study reported the results of the first clinical experience with DEvice©, a novel portable instrument that assesses the gaseous state of the tear film by detecting variations in humidity levels, aiming at discriminating patients with DED from healthy controls. Using DEvice©, patients with DED showed statistically significant higher values of RH compared to the control group. These results were consistent with data reported in a small observational pilot study, in which this tool was utilized to measure the fluctuation in relative humidity by analyzing the curvature shape, the speed of changes, and the ultimate RH after one minute [20]. Conversely, no statistically significant differences were detected for the mean values of temperature between the two groups. In contrast to healthy subjects, divergent findings have been documented regarding the ocular surface temperature following a blink in individuals with DED. A previous study reported that in patients with DED, subjective distress was experienced earlier than in the control group, which was correlated with decreased corneal temperatures and increased tear evaporation [25]. In our study, no temperature disparities were observed between the two groups under investigation.

An attempt at correlating values of RH detected with DEvice© with both subjective symptoms and objective parameters measured by Keratograph 5M was performed. A significant positive correlation was found between RH and OSDI score, while a mild trend of negative correlation, albeit not statistically significant, was found between RH and NIKBUT-first, the parameter that was most altered in DED patients. Moreover, the ROC curve showed that RH had a significant discriminating power to detect DED, being able to screen affected patients with a good sensitivity.

Relative humidity is a measure of the amount of water vapor in a mixture of water and air compared to the maximum amount of water vapor that may be present at a specific temperature [26]. The study device can detect fluctuations in RH values in a closed environment around the surface of the eye. An analogous method is used by dermatologists to quantify transepidermal water loss. The increase in transepidermal water loss is a characteristic feature of atopic dermatitis and can occur before the visible symptoms of the disease [27]. A previous study documented the validation and consistency of a modified dermatology instrument utilized to quantify the rate of tear evaporation. The study enrolled fifteen participants and comprised two stages: one in which they wore contact lenses and the other in which they did not. They found that lenses increased the variability and height of measurements and, due to the fact that increased humidity decreased the rate of evaporation, environmental regulation was crucial [17]. The tear film lipid layer represents a physiological indicator of tear film stability, and the tear evaporation rate can be used to classify subtypes of DED [1]. In 2021, a study evaluated the repeatability and reproducibility of measuring the rate at which the tear film evaporates using a handheld closed-chamber evaporimeter. A total of 40 individuals who were in good health and had no eye-related illnesses were selected to participate in this study. The VapoMeter offers consistent and accurate measurements of tear film evaporation that can be replicated [28]. Another study that analyzed the evaporation rate detected real-time changes in patients with obstructive meibomian gland dysfunction (MGD) compared to a control group. They observed an increase in the evaporation rate, which could be indicative of an unstable tear film in individuals with MGD [29]. According to this study, our results demonstrated higher RH value compared to controls.

Fluctuating temperature, humidity levels, seasonal variations, airflow speed, and pollutants are recognized as factors that might affect the tear film evaporation. These factors can either improve or worsen symptoms of DED [30,31,32]. The evaporation rate is affected by humidity; hence, it is important to monitor humidity while taking measurements. A previous study assessed the repeatability of tear evaporimetry to examine the impact of altering environmental humidity on the ocular surface temperature (OST) and tear evaporation rates (TERs) in a controlled adverse environmental (CAE) chamber. The TER was not changed significantly [33]. A categorization system for tear dysfunction has emphasized the influence exerted by evaporative systems that are susceptible to variations in temperature, humidity, and airflow [34]. Furthermore, Kimball at al. demonstrated that the rate of thinning in free air is higher than the stated rates of evaporation. This is because the preocular chambers employed in evaporimeters limit the movement of air over the tear film, resulting in reduced evaporation relative to the conditions of free air [35]. Moreover, additional elements, including the composition and structure of the lipid layer, play a significant role [36]. Peng at al. conducted a preliminary investigation including human subjects, which confirmed the possibility of using the flow evaporimeter to measure the tear film evaporation rates in vivo. They tested the new evaporimeter in 3 healthy male subjects on 15 different occasions. All subjects attended at approximately the same time of day. Before each measurement of tear evaporation, participants were instructed to spend at least 15 min in an air-conditioned room with a temperature of around 23 °C to adjust to the environment [37].

The instrument, similar to the ones previously examined, was noninvasive. Every tool presents a distinct sort of chamber to enclose the space surrounding the eye. One of these devices is equipped with swimming goggles that may be attached to assess the rate of evaporation on the eye and the surrounding skin [17,28]. Participants in a different study utilized flow-evaporimeter goggles [37]. Another method suggested assessing TERs involved using infrared thermography in a clinical room setting. Image sequences were collected and subsequently converted into a set of grayscale jpeg pictures. Following this, the TERs were calculated [33]. The DEvice©, compared to tools already existing on the market, is easier to use, as does not require goggles and it is positioned by the operator on the orbital margins of the patient, who is asked to avoid blinking during the assessment period. Simultaneously, the acquired information can be instantly displayed on the computer without the need for conversion, enabling the concurrent measurement of both relative humidity (RH) and temperature per second. At the same time, the instrument is both portable and more affordable.

Therefore, it is evident that using a sensor that can detect differences in RH among patients with different types and severities of DED and the healthy population has a significant advantage. Accurately identifying the signs of DED is crucial for determining the extent of the disease and starting the proper treatment. Although there have been significant advancements in technology and research focused on DED, many cases remain undiagnosed. A recent study identified at least one abnormal parameter in all patients who were scheduled for senile cataract surgery with no prior diagnosis of DED or established risk factors. Furthermore, 55% of the study participants were found to have undiagnosed DED [38]. The signs and symptoms of ocular surface dysfunction often have a limited association, rendering patient-reported symptoms or histories unreliable for appropriately assessing the state of the ocular surface [39].

Various all-in-one instruments can be employed to evaluate the features of the ocular surface for clinical and research purposes. This noninvasive evaluation overcomes the drawbacks of conventional tests using the instillation of dyes (e.g., fluorescein tear break-up time) that can perturbate the ocular surface and can suffer from the subjectivity of the readings [40]. Nevertheless, noninvasive automated instruments are frequently relatively expensive and not always available in all settings due to occupancy challenges. Therefore, many DED cases remain understudied. Portable and inexpensive devices could help with the wider adoption of DED screening in healthcare settings with limited access to high-tech imaging. For instance, a recent study proposed the use of a smartphone-attachable device for TMH calculation, whose results were in good agreement with the measurements obtained with conventional slit lamp examination [41].

Although this study represents the first investigation of the diagnostic accuracy of DEvice© for discriminating between DED patients and health subjects, it is crucial to recognize and deal with the inherent limitations that deserve mentioning. Firstly, the limited sample size could have hampered the possibility to reach statistical significance in the correlations between HR and the other ocular surface parameters, in particular NIKBUT-first. Secondly, our study did not analyze nor stratify DED according to subtype, severity, and treatment employed. At the same time, the discriminatory function has some limits, particularly on account of the sample size (40 patients in total) that was used here. Increasing the sample size could potentially improve the efficiency. In particular, only fair discrimination ability was demonstrated since the AUC for the RH was 0.78, and further studies with a larger number of subjects are necessary.

In conclusion, the tool DEvice©, which quantifies the humidity variations in a closed environment around the ocular surface, is a fast, portable, noninvasive, and inexpensive tool for effectively screening patients with DED. Further studies with larger sample sizes and more robust designs are needed to clarify its potential role in the DED diagnostic armamentarium.

## Figures and Tables

**Table 1:** Correlation matrix among RH, TMH, NIKBUT-first, NIKBUT-average, redness, and OSDI score.

		RH	TMH	NIKBUT-First	NIKBUT-Average	OSDI
RH	Pearson’s r	-				
	df	-				
	*p*-value	-				
TMH	Pearson’s r	−0.091	-			
	df	38	-			
	*p*-value	0.577	-			
NIKBUT-first	Pearson’s r	−0.295	−0.358	-		
	df	38	38	-		
	*p*-value	0.065	0.023	-		
NIKBUT-average	Pearson’s r	−0.101	−0.262	0.810	-	
	df	38	38	38	-	
	*p*-value	0.534	0.102	<0.001	-	
OSDI	Pearson’s r	0.406	0.241	−0.585	−0.425	-
	df	38	38	38	38	-
	*p*-value	0.009	0.134	<0.001	0.006	-

## Data Availability

The raw data supporting the conclusions of this article will be made available by the authors without undue reservation.
